# Friction Behaviors and Wear Mechanisms of Carbon Fiber Reinforced Composites for Bridge Cable

**DOI:** 10.3390/polym16233446

**Published:** 2024-12-09

**Authors:** Guijun Xian, Xiao Qi, Rui Guo, Jingwei Tian, Huigang Xiao, Chenggao Li

**Affiliations:** 1 Key Lab of Structures Dynamic Behavior and Control, Harbin Institute of Technology, Ministry of Education, Harbin 150090, Chinaguorui@hit.edu.cn (R.G.); xiaohg@hit.edu.cn (H.X.); lichenggao@hit.edu.cn (C.L.); 2 Key Lab of Smart Prevention and Mitigation of Civil Engineering Disasters, Harbin Institute of Technology, Ministry of Industry and Information Technology, Harbin 150090, China; 3 School of Civil Engineering, Harbin Institute of Technology, Harbin 150090, China; 4 Yangtze River Delta Carbon Fiber and Composite Material Innovation Center, Changzhou 213126, China; qixiao@ccicyd.com

**Keywords:** epoxy composites, carbon fiber, harsh service environment, friction behavior, wear mechanism

## Abstract

Carbon fiber reinforced epoxy resin composites (CFRP) demonstrate superior wear resistance and fatigue durability, which are anticipated to markedly enhance the service life of structures under complex conditions. In the present paper, the friction behaviors and wear mechanisms of CFRP under different applied loads, sliding speeds, service temperatures, and water lubrication were studied and analyzed in detail. The results indicated that the tribological properties of CFRP were predominantly influenced by the applied loads, as the tangential displacement generated significant shear stress at the interface of the friction pair. Serviced temperature was the next most impactful factor, while the influence of water lubrication remained minimal. Moreover, when subjected to a load of 2000 g, the wear rate and scratch width of the samples exhibited increases of 158% and 113%, respectively, compared to those loaded with 500 g. This observed escalation in wear characteristics can be attributed to irreversible debonding damage at the fiber/resin interface, leading to severe delamination wear. At elevated temperatures of 100 °C and 120 °C, the wear rate of CFRP increased by 75% and 112% compared to that at room temperature. This augmentation in wear was attributed to the transition of the epoxy resin from a glassy to an elastic state, which facilitated enhanced fatigue wear. Furthermore, both sliding speed and water lubrication displayed a negligible influence on the friction coefficient of CFRP, particularly under water lubrication conditions at 60 °C, where the friction coefficient was only 15%. This was because the lubricant properties and thermal management provided by the water molecules, which mitigated the frictional interactions, led to only minor abrasive wear. In contrast, the wear rate of CFRP at a sliding speed of 120 mm/s was found to be 74% greater than that observed at 60 mm/s. This significant increase can be attributed to the disparity in sliding rates, which induced uncoordinated deformation in the surface and subsurface of the CFRP, resulting in adhesive wear.

## 1. Introduction

Marine engineering construction and maintenance are foundational to establishing a strong maritime nation. However, in highly corrosive marine environments, traditional steel-reinforced concrete and steel structures face significant durability challenges. Particularly in high-temperature, high-humidity, high-load hygrothermal environments and under external impact loads, metal materials used in marine engineering structures are prone to corrosion [1,2], wear, and fatigue [3], severely threatening the service life of components or structures [3]. Therefore, there is an urgent need to develop long-life, highly durable materials that can safely serve under high-temperature, high-humidity, high-load, and reciprocating sliding speeds.

Fiber reinforced polymer (FRP) has become an important engineering material [4]. FRP composites consist of fibers, a resin matrix, and a fiber–resin interface [5,6,7,8]. Among them, carbon fiber reinforced polymer (CFRP) possesses advantages such as light weight, high strength, and corrosion resistance [3,9]. Since the end of the last century, they have been gradually applied in bridge engineering structures. For example, in 1996, the world’s first pedestrian cable-stayed bridge entirely made of CFRP was constructed in Tsukuba, Japan, greatly enhancing the safety and reliability of bridges. Therefore, the application of CFRP in marine engineering structures holds significant research importance.

However, CFRP experiences asynchronous deformation between the carbon fibers and the epoxy resin matrix during the service process, leading to defects at the fiber/resin interface and within the resin matrix, generating the inevitable types of damage (such as fiber breakage, resin matrix cracking, and fiber/resin interface debonding) [10,11]. Additionally, compared to carbon fibers, the epoxy resin matrix is brittle, prone to cracking, and has poor impact and weather resistance [6,12]. This causes the fiber/resin interface to de-bond or the resin matrix to crack before fiber breakage occurs [13]. Therefore, the service life of CFRP is jointly controlled by the fiber/resin interface and the epoxy resin matrix.

The carbon fiber/epoxy resin interface acts as a “bridge” connecting the carbon fibers and the epoxy resin matrix [14]. However, during the tribological processes of CFRP, microcracks and other defects easily form along the fiber direction at the carbon fiber/epoxy resin interface when transferring loads, preventing the uniform transmission of external loads between the fibers and resin matrix, significantly affecting the service performance and life of CFRP in applications [15]. Moreover, microcracks within the epoxy resin matrix often serve as initiation points for the decline in CFRP’s mechanical properties. Particularly under the combined effect of external loads and service environments, these microcracks within the resin matrix begin to initiate, propagate, and gradually develop into macroscopic cracks, leading to interlayer failure phenomena in CFRP and eventually evolving into overall failure [16,17]. Furthermore, as actual service conditions continue to deteriorate, especially under the continuous friction of seawater in deep-sea environments, ongoing wear by solid materials, and alternating thermal and humid loads [18], irreversible damage is likely to occur at the fiber/resin interface and within the resin matrix. This ultimately leads to changes in the microstructure and morphology of CFRP during friction, corresponding to different wear mechanisms [19]. Therefore, it is imperative to clarify the friction behaviors and wear mechanisms of CFRP under various service conditions.

During the tribological processes under different service conditions, CFRP undergoes creep, yield, twist, and deformation changes, ultimately manifested macroscopically as the detachment and removal of wear debris [20,21]. This macroscopic mechanism of material surface wear debris removal constitutes an extremely complex thermodynamic process, closely related to the fiber/resin interface bonding, the physical and chemical properties of the resin matrix, and the service environment [22,23]. The applied loads, sliding speeds, service temperatures, and water lubrication in actual service conditions significantly impact the wear life of CFRP [24,25]. This is because different service conditions determine whether a lubricating transfer film can form during the steel ball-CFRP friction process, which ultimately plays a critical role in the friction behavior and wear mechanisms of composites [26]. Additionally, to provide a semi-quantitative explanation of the lubricating transfer film, the line roughness (LR) is introduced, representing the average roughness at each point along the scratch direction, being used to characterize the microscopic morphology of the lubricating transfer film along the wear track. This helps to visualize the tribological performances of CFRP. Therefore, studying the friction behavior and wear mechanisms of CFRP under different applied loads, sliding speeds, service temperatures, and water lubrication holds significant scientific and engineering value.

In summary, to clarify the friction behaviors and wear mechanisms of CFRP used in marine engineering under different service environments and to elucidate the crucial roles of carbon fibers and epoxy resin during the friction process, this paper investigates the tribological performance of CFRP under various applied loads (500, 1000, 1500, 2000 g), sliding speeds (60, 80, 100, 120 mm/s), service temperatures (R.T., 60, 80, 100, 120 °C), and water lubrication environments (R.T., 60, 80, 95 °C). Using microscopic characterization techniques such as scanning electron microscopy and super-depth microscopy, the wear resistance mechanisms are revealed. Additionally, a sensitivity analysis and comparison of the factors affecting the tribological performance of CFRP under each service environment are conducted, aiming to lay the theoretical foundation for the application of CFRP in actual hygrothermal environments.

## 2. Materials and Methods

### 2.1. Raw Materials and Sample Preparation

**Figure 1:**
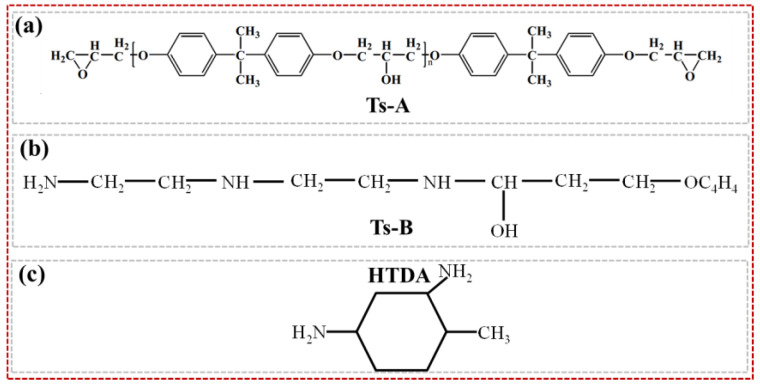
Epoxy resin matrix and its corresponding two curing agents: (**a**) epoxy Ts-A; (**b**) epoxy Ts-B; (**c**) curing agent HTDA.

In this study, the large-tow carbon fiber was produced by Sinopec Shanghai Petrochemical Co., Ltd. (48 K, Shanghai, China). Bisphenol A-type epoxy resin Ts-A and its corresponding curing agent Ts-B were used. To achieve room temperature (R.T.) curing and complete post-curing at high temperatures, another amine curing agent, HTDA (4-methylcyclohexane-1,3-diamine), was also used in combination. The weight ratios of these three components were 100:16.3:10.9, and their specific chemical structures were shown in Figure 1. The Ts epoxy resin system was produced by Shandong Dagong Composite Materials Co., Ltd. (Ts, Linyi, China), with a viscosity and density of 2000 cPs and 1200 kg/m^3^, respectively.

**Figure 2:**
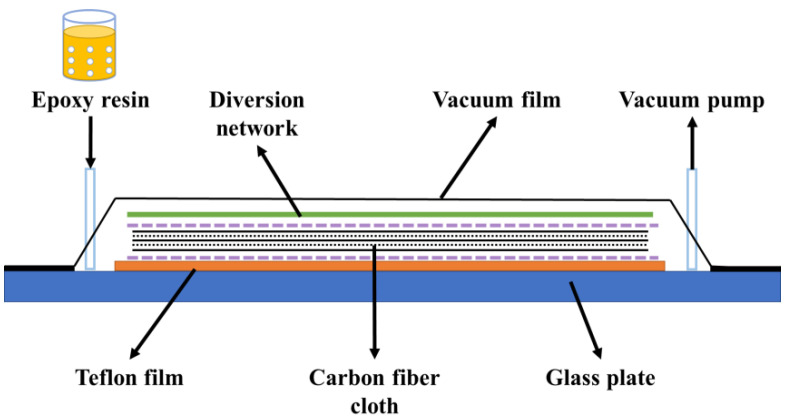
Vacuum-assisted resin injection molding process diagram.

CFRP was prepared using the vacuum-assisted resin infusion molding method, as shown in Figure 2. The specific steps were as follows: Place a polytetrafluoroethylene sheet and a release fabric sequentially on a glass plate, stack six layers of carbon fiber fabric in the same fiber direction on the release fabric, and place another release fabric and a flow mesh on top of the fiber fabric. Then, seal the assembly in a vacuum bag. The epoxy resin (Ts-A group) and two curing agents (Ts-B and HTDA) were mixed uniformly using a homogenizer (ZYMC350VS, Shenzhen Zhongyi Technology Co., Ltd., Shenzhen, China) at 5000 rpm for 5 min, followed by ultrasonic vibration to eliminate internal bubbles. Using a vacuum pump, the mixed epoxy resin was infused into the carbon fiber fabric. After curing at R.T. for 48 h, the mold was removed, and the composites were post-cured in an oven at 60 °C for 24 h to obtain the CFRP plate.

### 2.2. Friction and Wear Testing

**Figure 3:**
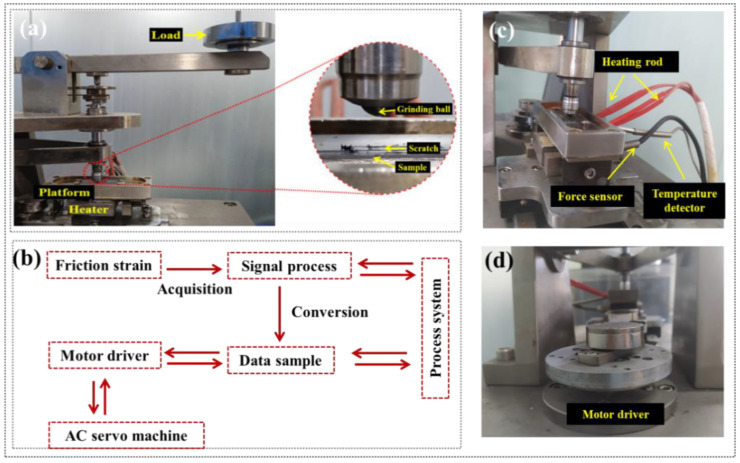
Reciprocating friction and wear testing: (**a**) friction pair system; (**b**) machine structure principle; (**c**) friction disk system; (**d**) motor driver.

The friction and wear performances of the CFRP were tested using a reciprocating tribology machine (HRTA02, Jinan HengXu Testing Machine Technology Co., Ltd., Jinan, China). The sample dimensions were 36 mm (length) × 10 mm (width) × t mm (thickness), and a Q235 steel ball with a diameter of 6.35 mm was used as the counter surface, as shown in Figure 3a. It was noted that the reciprocating friction direction was along the direction of the fiber, which was convenient to accurately evaluate the uniform stress transfer at the carbon fiber/epoxy resin interface during friction. When the sample surface underwent reciprocating friction motion, the friction force acted as an internal force, causing slight deformation of the support platform. By detecting the micro-deformation stress of the elastic support mechanism, the strain signal was processed through a signal conditioning module to remove noise, amplify it, and convert it into an alternating friction force. The acquisition system collected the data and sent it to a computer. The real-time coefficient of frictions (COFs) during the friction process were obtained by calculating the ratio of the alternating friction force to the normal pressure generated by the weights, as illustrated in Figure 3b–d. The wear rate (Ws) was calculated from the volume loss of the material before and after friction, using the following equation:

(1) $$Ws=\frac{\Delta V}{lF}\left({\mathrm{mm}}^{3}/N\cdot m\right)$$

where Δ*V* is the sample volume change; *F* is the normal load; *l* is the total length of the friction process.

Each test was set to a reciprocating cycle of 10,000 times. Additionally, to comprehensively verify the friction and wear performance of CFRP, the service conditions were set to different applied loads (500, 1000, 1500, 2000 g), sliding speeds (60, 80, 100, 120 mm/s), service temperatures (R.T., 60, 80, 100, 120 °C), and water lubrication (R.T., 60, 80, 95 °C). The effects of sliding speeds, service temperatures, and water lubrication on friction behavior and wear rate of CFRP were studied, and the applied load was all 500 g. Among them, it was noted that the water lubrication condition required the addition of deionized water to the sample tank in Figure 3c and ensured that the water surface height was greater than the sample thickness so that the scratch contact surface was always submerged by water. When operating at elevated temperatures (80 °C and 95 °C), water needed to be added regularly during the test to ensure adequate water lubrication during friction.

### 2.3. Morphology Analysis

After the friction and wear tests, the surfaces of the CFRP samples were attached to aluminum sheets using conductive adhesive. The sample surfaces were then gold-sputtered using a gold sputter coater (E5200, Bio-Rad, Cambridge, UK). Surface morphology analysis of the samples was conducted using a scanning electron microscope (SEM, VEGA3 TESCAN, Washington, DC, USA) in secondary electron imaging mode with an operating voltage of 30 kV.

To study the effects of different service environments on the friction behavior and wear mechanisms of CFRP, a three-dimensional super-depth microscope (OLYMPUS DSX500, Tokyo, Japan) was used to analyze the sample scratch areas and the corresponding morphology of the grinding ball. In continuous scanning mode, quantitative measurements were performed on approximately 8 mm of the middle region of the scratch. The scratch length and wear scar width (WSW) were averaged from 10 sets of data to calculate the wear rate.

## 3. Results and Discussion

### 3.1. Friction Behaviors

#### 3.1.1. Effect of Applied Loads

In general, the friction force inside the friction pair system is positively correlated with the applied load, independent of the friction contact area. However, for polymers and their composites, when the load is high, the friction force is not only related to the applied load but also the contact area [27]. Therefore, to study the friction behaviors and wear morphologies, Table 1 summarizes the coefficients of frictions (COFs), wear rates, and corresponding average line roughness (LR) values of CFRP under various applied loads.

As the applied load increased, the COFs fluctuation range of CFRP initially increased and then decreased. This was because the effect of the load on COFs was achieved by changing the actual contact area. Under lower loads (500 g), the contact state at the friction pair was in an elastic or viscoelastic state, resulting in the actual contact area being proportional to the applied load. Therefore, the contact at the sample/grinding ball interface was a direct point contact, resulting in the highest COFs for the sample. Under higher loads (2000 g), the contact state between the grinding ball and CFRP changed from elastic or viscoelastic to plastic or viscoplastic [28]. At the time, excessive plastic deformation increased the interface contact area, causing the CFRP COFs to no longer change significantly with increasing load. However, higher loads would lead to the increased wear and friction temperature on the CFRP surface, generating a lot of debris in the friction track area. For example, the Ws and WSW of the CFRP sample under a 2000 g load increased by 157.9% and 112.6% compared to a 500 g, respectively, indicating severe wear on the CFRP surface. This demonstrated that high-load conditions had a significant impact on the tribological performance of fiber composites. This was because, under reciprocating and high shear stress, the difference in deformation resistance between fibers and the resin matrix caused damage such as debonding at the fiber/resin interface, reducing the strength and stiffness of CFRP to withstand external loads.

However, it was noteworthy that even under a 2000 g load, the LR value of CFRP was only 15.9 µm, indicating that the wear loss on the wear scar surface was relatively uniform, without large-scale spalling. This was because the uneven surface formed by carbon fibers during friction easily became frictional hotspots, causing resin debris to stick together and preventing further scratching at the friction contact interface.

**Figure 4:**
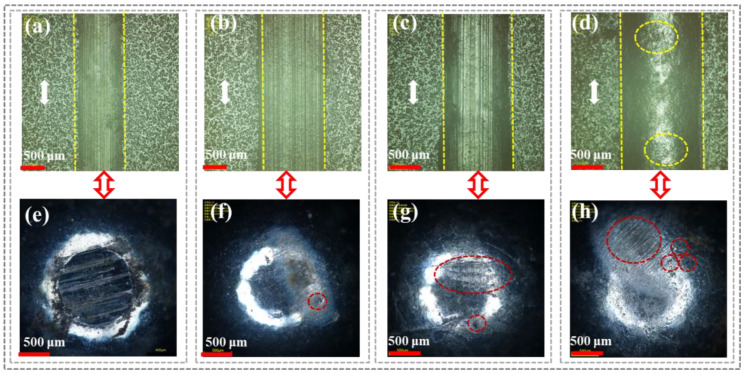
CFRP surface scratches and corresponding micromorphology of grinding ball under different loads: (**a**,**e**) 500 g; (**b**,**f**) 1000 g; (**c**,**g**) 1500 g; (**d**,**h**) 2000 g.

Figure 4 shows the surface wear tracks of CFRP under different loads and the corresponding microscopic morphology of the grinding ball surfaces to analyze the friction behavior and wear degree during service. As shown in Figure 4a (500 g) and Figure 4b (1000 g) under low loads, there were no obvious scratches on the wear track surface of CFRP. This was because the resin on the CFRP surface was preferentially worn and formed a transfer film under continuous load pressure. Additionally, the graphite in the carbon fibers provided a lubricating effect due to easy sliding between the layers. Therefore, under low loads, CFRP only experienced slight adhesive wear and abrasive wear. This was further evidenced by the absence of significant wear marks on the corresponding grinding ball surfaces under these two load conditions (Figure 4e,f), which was attributed to the fact that the transfer film mitigated severe friction and wear at the sample/grinding ball interface.

In comparison, as the applied load increased to 1500 g (Figure 4c) and 2000 g (Figure 4d), the transfer film surface in the scratch area of CFRP gradually developed microcracks, especially under a 2000 g load. A possible reason was that the high load changed the contact state at the sample/grinding ball interface from elastic or viscoelastic to plastic or viscoplastic, ultimately causing plastic deformation of the resin matrix. Furthermore, under high loads (1500 g and 2000 g), many parallel wear marks appeared on the grinding ball surface (Figure 4g,h), indicating more severe friction and wear at the CFRP/grinding ball interface. This was because the transfer film composed of resin debris could not continue to withstand high shear stress under high loads and got damaged, exposing the carbon fibers. The high surface hardness of the carbon fibers led to sudden breakage, increasing friction resistance and mechanical obstruction against the grinding ball.

#### 3.1.2. Effect of Sliding Speeds

The sliding speed of the grinding ball significantly affected the friction behavior and wear rate of CFRP during service, particularly as high sliding speeds could cause excessive plastic deformation of the resin matrix and even rapid displacement of material from the wear area.

As shown in Table 2, with the increase in sliding speed, the fluctuations in COFs of CFRP were not significant, indicating relatively stable friction forces between the tribo-pairs during sliding. However, the Ws and WSW of CFRP increased by 78.2% and 39.6% compared to the lower sliding speed (60 mm/s), respectively, under the high sliding speed (120 mm/s). This indicated that although the COFs were relatively similar, the wear rates significantly increased under both low and high sliding speed conditions. This was because the greater speed differential at the sample/grinding ball interface caused the different deformation of the sample subsurface and surface, increasing material loss on the CFRP surface and causing intense friction between carbon fiber fragments and the grinding ball, thereby increasing the wear loss. Additionally, it was noteworthy that at a sliding speed of 100 mm/s, the LR values of the CFRP surface showed minimal fluctuation, as the resin debris encapsulated the thinning, cracking, and breaking carbon fibers, preventing their tips from being exposed on the scratch surface and alleviating the friction degree at the CFRP/grinding ball interface.

**Figure 5:**
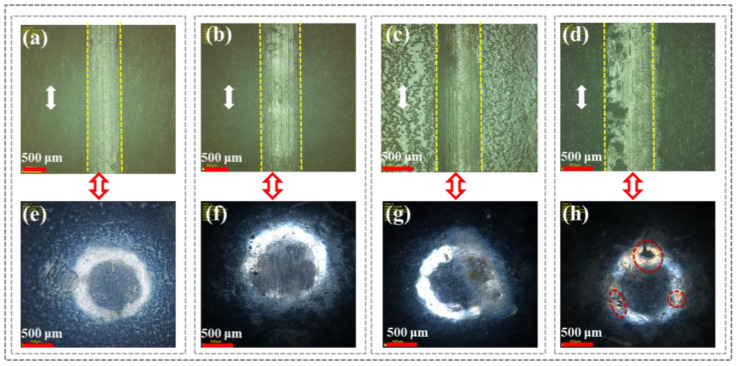
CFRP surface scratches and corresponding micromorphology of grinding ball under different sliding speeds: (**a**,**e**) 60 mm/s; (**b**,**f**) 80 mm/s; (**c**,**g**) 100 mm/s; (**d**,**h**) 120 mm/s.

Figure 5 shows the wear morphologies of the CFRP and grinding ball surfaces under different sliding speeds. As the sliding speed increased, Figure 5a–d revealed that wear marks parallel to the sliding direction appeared on the CFRP scratch surface. Notably, at sliding speeds of 100 mm/s (Figure 5c) and 120 mm/s (Figure 5d), wear debris were clearly visible on the surface. This was because a higher sliding speed would make the shear rate larger, resulting in a lot of frictional heat in the sample/grinding ball interface area, causing the epoxy resin matrix to gradually soften because of insufficient heat dissipation, ultimately resulting in slight abrasive wear and adhesive wear. Therefore, higher shear sliding speeds increased the wear volume of CFRP, leading to intense friction and wear at the sample/grinding ball interface. This was because the epoxy resin matrix underwent excessive plastic deformation at higher sliding speeds, causing continuous compression of carbon fiber debris and increasing the temperature of the friction interface, which ultimately resulted in various shapes of spots appearing on the grinding ball surface (Figure 5e–h).

#### 3.1.3. Effect of Service Temperatures

The service temperature of epoxy resin-based composites is a critical factor affecting the friction and wear performance of polymer composites. Particularly under high-temperature service conditions, due to the continuous reciprocating friction, the friction heat generated inside the friction system had no time to transfer to the epoxy resin subsurface, resulting in higher temperatures in the contact area and ultimately causing significant plastic deformation. Table 3 shows the friction behavior and wear rate of CFRP at different service temperatures. As the service temperature increased (R.T. to 120 °C), the COFs of CFRP showed a decreasing trend, especially at 120 °C, where the average COFs decreased by 37.5% compared to room temperature. This was because the high temperature of 120 °C was close to the Tg of CFRP (136.8 °C), causing substantial plastic deformation of the resin matrix, which alleviated the frictional resistance between the grinding ball and CFRP. However, excessive plastic deformation under high-temperature conditions led to a significant increase in wear volume during friction. For example, compared to room temperature friction, the Ws and WSW of CFRP at 120 °C increased by 112.2% and 48.8%, respectively. This was because the higher service temperature (120 °C) caused the polymer surface to change from a glassy state to a highly elastic or viscoelastic state, forming a low-viscosity fluid layer in the scratch area, resulting in a very low coefficient of friction for CFRP under high-temperature service conditions but a significantly increased wear volume. This could also be verified from the gradually increasing LR values.

**Figure 6:**
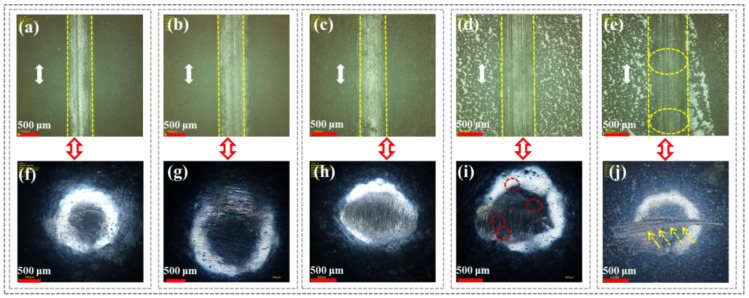
CFRP surface scratches and corresponding micromorphology of grinding ball under different serviced temperatures: (**a**,**f**) R.T.; (**b**,**g**) 60 °C; (**c**,**h**) 80 °C; (**d**,**i**) 100 °C; (**e**,**j**) 120 °C.

Figure 6 shows the scratch and corresponding grinding ball surface morphology of CFRP under different service temperatures. At lower service temperatures (R.T., 60 °C, and 80 °C), no obvious scratch damage, fiber cracking, and fragmentation were observed in the wear areas of CFRP (Figure 6a–c). This was because the excellent thermal conductivity of carbon fibers during friction prevented heat accumulation at the fiber/resin interface due to frictional heating, allowing the epoxy resin matrix to remain in a glassy state and retain surface hardness. In contrast, as the service temperature increases to 100 °C (Figure 6d) and 120 °C (Figure 6e), more wear scratches appeared on the CFRP surface, with localized plastic deformation and grooves particularly evident at 120 °C. Meanwhile, compared to low-temperature service conditions (Figure 6f–h), significant wear marks appeared on the grinding ball surface at 100 °C (Figure 6i) and 120 °C (Figure 6j). However, it was noteworthy that spot marks (red circles) only appeared on the grinding ball surface at 100 °C, and a possible reason was that the glassy resin matrix changed to a high elastic state, exposing high-modulus carbon fiber debris encapsulated by the resin matrix, which directly contacted and frictionally interacted with the grinding ball.

#### 3.1.4. Effect of Water Lubrication

Based on the aforementioned dry-state friction analysis, the wet-state friction behavior of CFRP under different temperature water lubrication conditions was selected for comparison to verify its friction and wear performance under harsh conditions. It was important to note that the friction behavior and wear rate of CFRP under wet-state friction were analyzed by the coupling effect of the liquid water and solid lubrication films compared to dry-state friction. At the same time, the continuity and integrity of the liquid water films were related to the service temperature during the friction process. Therefore, four operating temperatures (R.T., 60 °C, 80 °C, and 95 °C) were selected for the tribological performance tests of the samples. Here, 95 °C was chosen for the water lubrication condition to prevent water molecules from boiling and rapidly evaporating at 100 °C.

As shown in Table 4, with the continuous increase in operating temperature, the average COFs of CFRP gradually increased, especially under the 95 °C water lubrication condition. This was because, at 95 °C (close to the boiling point of water), water molecules transition from a liquid to a gaseous state, leading to discontinuity in the water molecule film at the sample/grinding ball interface. Moreover, compared to dry friction (under different applied loads, different sliding speeds, and different service temperatures), CFRP exhibited better wear resistance under different temperature water lubrication friction conditions. For example, compared to a 2000 g load (Table 1), 120 mm/s sliding speed (Table 2), and 120 °C dry-state friction (Table 3), the Ws and WSW of CFRP under the 95 °C water lubrication (Table 4) decreased by 58.3% and 41.6%, 45.4% and 31.5%, and 33.1% and 25.5%, respectively. Additionally, the LR values of CFRP under different temperature water lubrication conditions showed little difference. For example, compared to room temperature water lubrication, the LR value of CFRP under the 95 °C water lubrication condition increased by only 16.5%. This was because the water molecule film not only provided lubrication but also resisted wear scratches caused by the grinding ball. For example, Zhang et al. [29] reported that when the tribo-pair was immersed in a seawater condition, seawater acted as a lubricant while increasing the friction distance between the material and the counter surface, mitigating the wear degree in the interface region and changing the wear mechanism from severe fatigue wear to mild abrasive wear during friction.

**Figure 7:**
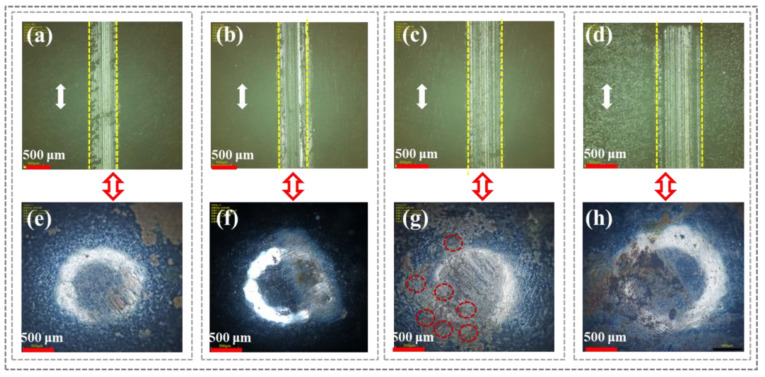
CFRP surface scratches and corresponding micromorphology of grinding ball under different water lubrication: (**a**,**e**) R.T.; (**b**,**f**) 60 °C; (**c**,**g**) 80 °C; (**d**,**h**) 95 °C.

Figure 7 shows the scratch marks on the CFRP surface and the corresponding grinding ball’s microscopic morphology under different temperature water lubrication conditions. As shown in Figure 7a–d, compared to lower operating temperatures (R.T. and 60 °C), significant wear marks appeared along the sliding direction on the CFRP surface under high-temperature (80 °C and 95 °C) water lubrication conditions. This was because the transfer film of resin debris inside CFRP was destroyed by the rapid evaporation of water molecules, reducing the distance between the CFRP/grinding ball tribo-pair and causing direct contact friction. This was attributed to two reasons: firstly, the increased temperature enhanced the movement of epoxy resin molecular chains and reduced the surface hardness of CFRP; secondly, the higher temperature gave water molecules more kinetic energy, causing continuous evaporation and boiling, thereby disrupting the water film continuity and increasing the friction contact area between CFRP and the grinding steel ball. Additionally, as shown in Figure 7e (R.T.) and 7f (60 °C), no significant friction scratches were observed on the grinding ball surface, only electrochemical corrosion marks. However, as the operating temperature increased to 80 °C and 95 °C, numerous corrosion spots (marked by red circles) appeared on the grinding ball surface (Figure 7g,h), indicating that the dense oxide film on the steel surface was worn away through direct contact friction, leading to severe corrosion wear.

### 3.2. Wear Mechanism Analysis

**Figure 8:**
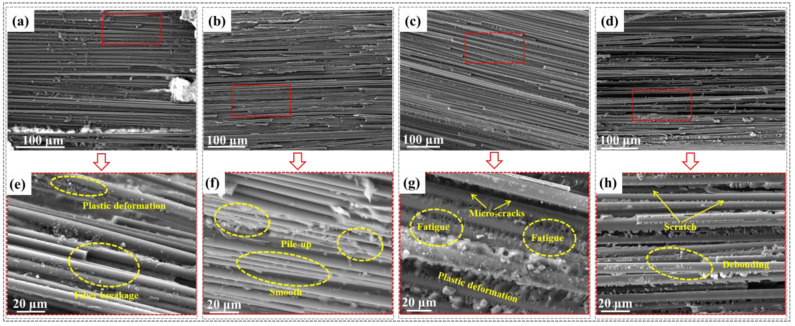
Wear morphology comparison of CFRP under each of the most severe service conditions: (**a**,**e**) 2000 g applied load; (**b**,**f**) 120 mm/s sliding rate; (**c**,**g**) 120 °C serviced temperature; (**d**,**h**) 95 °C water lubrication.

To comprehensively compare the mechanisms influencing the friction behavior and wear rate of CFRP under different service conditions, Figure 8 summarizes the microscopic wear morphology of the CFRP surface under applied load (2000 g), sliding speed (120 mm/s), service temperature (120 °C), and high-temperature (95 °C) water lubrication. Figure 8a–d show the microscopic morphology of CFRP scratch areas under these hygrothermal conditions, revealing the wear evolution mechanism through micrographs of the resin matrix wear state and fiber/resin interface debonding distribution.

Regarding the influence of applied loads (Figure 8a), the wear degree of CFRP shows a certain layered structure, indicating that the high-load grinding ball generated significant shear stress in the interface area, leading to intense mechanical interlocking in the contact zone. Further, from the magnified image (Figure 8e), it was evident that the resin matrix had undergone excessive plastic deformation, and many fibers had been pulled out. This was because the high load on the CFRP surface significantly increased frictional resistance, causing large tangential displacements along the sliding direction, which destroyed the fiber/resin interface bonding. Meanwhile, the high load condition raised the friction contact interface temperature, causing excessive plastic deformation or even fracture of the protruding areas on the CFRP surface due to frictional heat. Ultimately, the plowed grooves on the CFRP surface were reshaped or cut, leading to larger Ws, WSW, and LR values under the 2000 g load condition (Table 1), consistent with the delamination wear mechanism [30,31,32].

Regarding the influence of sliding speeds (Figure 8b), the wear surface of CFRP was relatively intact, with the resin matrix uniformly encapsulating the carbon fibers, mitigating further scratches caused by shear stress. Meanwhile, from the magnified image (Figure 8f), it was clear that the fiber/resin interface within CFRP was well-bonded without debonding. Additionally, the resin matrix surface showed ripples, and wear debris was stacked, causing excessive plastic deformation on the CFRP surface, while the subsurface elastic deformation lags. Thus, the Ws, WSW, and LR values of CFRP remained relatively stable under high sliding speed service conditions (Table 2), consistent with the adhesive wear mechanism [30,33].

Regarding the influence of service temperatures (Figure 8c), although a resin debris transfer film forms on the CFRP surface during friction, its adhesion strength with the subsurface material was low. Furthermore, from the magnified image (Figure 8g), microcracks appeared on the CFRP surface. A possible reason was the high service temperature reduced CFRP’s resistance to thermal deformation, leading to a decrease in surface hardness. Additionally, at high service temperatures (close to Tg), the activity of the polymer chains in the epoxy resin matrix increased, resulting in inconsistent deformation across the wear track regions, causing cracks to initiate and propagate on the CFRP surface [34], ultimately developing into tearing and surface fatigue phenomena, consistent with the fatigue wear mechanism [30,35].

Regarding the influence of 95 °C water lubrication (Figure 8d), the lubrication and cooling functions of the water molecule film at the CFRP/steel ball interface resulted in a relatively smooth wear surface of CFRP, indicating significant resistance to reciprocating friction and high shear stress. Furthermore, Figure 8h shows parallel wear scratches on the CFRP surface along the sliding direction. However, the scratches were shallow, hardly causing excessive plastic deformation of the epoxy resin matrix. This explained the reason for the minimal Ws, WSW, and LR values of CFRP under the 95 °C water lubrication condition (Table 4). Simultaneously, during friction, the grinding steel ball caused plowing, cutting, cracking, and damage to the CFRP surface, resulting in parallel “furrow” slip lines, consistent with the abrasive wear mechanism [30,36].

In summary, among the factors influencing the friction coefficient and wear rate of CFRP, the applied load was the most sensitive, as tangential displacement caused significant shear stress at the sample and grinding ball interface; the service temperature was second; the sliding speed was third; and due to the lubrication and cooling functions of the water molecule film, high-temperature (95 °C) water lubrication was the least sensitive.

## 4. Conclusions

In the present study, the friction behaviors of carbon fiber reinforced composites (CFRP) were meticulously analyzed and compared across various parameters, including applied loads (500, 1000, 1500, and 2000 g), sliding speeds (60, 80, 100, and 120 mm/s), service temperatures (R.T., 60, 80, 100, and 120 °C), and water lubrication conditions (R.T., 60, 80, and 95 °C). Furthermore, the mechanisms underlying wear resistance were elucidated through advanced microscopic characterization techniques, including scanning electron microscopy and super-depth microscopy. Lastly, a sensitivity analysis was performed to evaluate and compare the influential factors affecting the friction behaviors and wear mechanisms of CFRP under the most challenging service environments, as evidenced by the observed changes in wear morphologies. Based on the test results and analysis, the following conclusions are drawn:

(1) The friction and wear performance of CFRP exhibited the greatest sensitivity to the applied load, as the tangential displacement generated considerable shear stress at the interface between the sample and the grinding ball. Service temperature emerged as the second most critical factor influencing these properties, while water lubrication ranked last due to its effective lubricating properties and heat dissipation capabilities.
(2) Compared to 500 g, the Ws and WSW of CFRP under a 2000 g load increased by 158% and 113%, respectively. This was due to the large tangential displacement along the sliding direction, causing debonding damage at the fiber/resin interface and reducing the strength and stiffness of CFRP against external loads. The wear mechanism was delamination wear.
(3) Compared to room temperature, the wear rate of CFRP at service temperatures of 100 °C and 120 °C increased by 75% and 112%, respectively. This was because the elevated temperature conditions made the glassy resin into a high elastic state, resulting in the continuous initiation and expansion of CFRP surface cracks, eventually generating tearing and surface fatigue phenomena, consistent with the fatigue wear mechanism.
(4) The sliding speed and water lubrication had an insignificant effect on the COFs of CFRP (less than 20%), especially under 60 °C water lubrication, where the COFs fluctuated by only 15%. This was because the lubricating and cooling functions of water molecules alleviated the friction state in the friction interface, causing only slight abrasive wear. The wear rate of CFRP at 120 mm/s sliding speed increased by 74% compared to 60 mm/s, due to the large speed differential causing uncoordinated deformation, triggering the adhesive wear mechanism.

## Figures and Tables

**Table 1:** Effects of applied loads on the friction coefficient and wear rate of CFRP.

Applied Load (g)	COFs	Ws (10^−6^ mm^3^/(N·m))	WSW (μm)	Maximum Wear Depth (μm)
500	0.04–0.12	15.2 (±2.2)	626.3 (±48.1)	8.7 (±1.4)
1000	0.05–0.10	26.7 (±1.9)	1012.5 (±39.6)	11.5 (±0.9)
1500	0.03–0.09	33.1 (±3.4)	1125.6 (±54.1)	13.3 (±1.2)
2000	0.03–0.07	38.9 (±2.4)	1321.8 (±44.2)	15.9 (±0.8)

Note: COFs were coefficients of friction; Ws was the wear rate; WSW was wear scar width; Maximum wear depth was the maximum of line roughness values.

**Table 2:** Effects of sliding speeds on friction coefficient and wear rate of CFRP.

Sliding Rate (mm/s)	COFs	Ws (10^−6^ mm^3^/(N·m))	WSW (μm)	Maximum Wear Depth (μm)
60	0.04–0.07	13.6 (±1.3)	659.2 (±21.3)	9.5 (±0.9)
80	0.04–0.05	18.2 (±2.0)	792.1 (±40.3)	10.1 (±1.3)
100	0.03–0.06	20.4 (±1.4)	864.3 (±36.0)	10.4 (±0.4)
120	0.05–0.09	24.2 (±1.7)	1037.5 (±38.4)	11.0 (±0.5)

**Table 3:** Effects of service temperatures on friction coefficient and wear rate of CFRP.

Serviced Temperature (°C)	COFs	Ws (10^−6^ mm^3^/(N·m))	WSW (μm)	Maximum Wear Depth (μm)
R.T.	0.06–0.09	14.2 (±1.4)	757.4 (±52.4)	10.8 (±0.9)
60	0.05–0.08	16.3 (±1.8)	781.6 (±46.9)	11.2 (±0.6)
80	0.05–0.08	21.2 (±3.6)	842.4 (±44.1)	11.5 (±1.2)
100	0.05–0.07	24.5 (±2.7)	931.6 (±39.2)	12.0 (±1.1)
120	0.04–0.06	29.7 (±2.0)	1127.3 (±35.6)	12.4 (±1.6)

**Table 4:** Effects of water lubrication on friction coefficient and wear rate of CFRP.

Water Lubrication (°C)	COFs	Ws (10^−6^ mm^3^/(N·m))	WSW (μm)	Maximum Wear Depth (μm)
R.T.	0.04−0.06	6.1 (±0.9)	554.2 (±19.8)	7.9 (±0.9)
60	~0.06	10.3 (±1.1)	595.8 (±32.7)	8.4 (±1.3)
80	0.05−0.07	14.8 (±1.2)	689.5 (±29.4)	8.7 (±0.4)
95	0.04−0.08	16.2 (±2.1)	773.6 (±43.5)	9. 2 (±0.8)

## Data Availability

The original contributions presented in this study are included in the article. Further inquiries can be directed to the corresponding author.
